# Investigating an increase in Florida manatee mortalities using a proteomic approach

**DOI:** 10.1038/s41598-021-83687-y

**Published:** 2021-02-19

**Authors:** Rebecca Lazensky, Cecilia Silva-Sanchez, Kevin J. Kroll, Marjorie Chow, Sixue Chen, Katie Tripp, Michael T. Walsh, Nancy D. Denslow

**Affiliations:** 1 Department of Physiological Sciences, Center for Environmental and Human Toxicology, University of Florida, Building 471, Mowry Road, PO Box 110885, Gainesville, FL 32611 USA; 2 Aquatic Animal Health Program, College of Veterinary Medicine, University of Florida, 2015 SW 16th Ave, PO Box 100136, Gainesville, FL 32608-0136 USA; 3 Proteomics and Mass Spectrometry, Interdisciplinary Center for Biotechnology Research, University of Florida, 2033 Mowry Rd, Gainesville, FL 32610 USA; 4 Department of Biology, University of Florida Genetics Institute, Gainesville, FL 32611 USA; 5 Save the Manatee Club, 500. N Maitland Ave., Maitland, FL 32751 USA; 6 Department of Biochemistry and Molecular Biology, University of Florida, Gainesville, FL USA

**Keywords:** Biotechnology, Ecology, Systems biology, Environmental sciences, Biomarkers

## Abstract

Two large-scale Florida manatee (*Trichechus manatus latirostris*) mortality episodes were reported on separate coasts of Florida in 2013. The east coast mortality episode was associated with an unknown etiology in the Indian River Lagoon (IRL). The west coast mortality episode was attributed to a persistent *Karenia brevis* algal bloom or ‘red tide’ centered in Southwest Florida. Manatees from the IRL also had signs of cold stress. To investigate these two mortality episodes, two proteomic experiments were performed, using two-dimensional difference in gel electrophoresis (2D-DIGE) and isobaric tags for relative and absolute quantification (iTRAQ) LC–MS/MS. Manatees from the IRL displayed increased levels of several proteins in their serum samples compared to controls, including kininogen-1 isoform 1, alpha-1-microglobulin/bikunen precursor, histidine-rich glycoprotein, properdin, and complement C4-A isoform 1. In the red tide group, the following proteins were increased: ceruloplasmin, pyruvate kinase isozymes M1/M2 isoform 3, angiotensinogen, complement C4-A isoform 1, and complement C3. These proteins are associated with acute-phase response, amyloid formation and accumulation, copper and iron homeostasis, the complement cascade pathway, and other important cellular functions. The increased level of complement C4 protein observed in the red tide group was confirmed through the use of Western Blot.

## Introduction

In 2013, 830 manatee deaths were reported in Florida. Based on an aerial survey performed in 2011, which estimated a population of 4834 manatees statewide, the deaths represented an estimated 17% loss of the Florida manatee population. Average manatee deaths per year have steadily increased from under 50 per year in the 1970’s to an average of about 400 per year by 2011^1^. The loss of 830 manatees in 2013 was a significant increase over baseline. On Florida’s east coast, the Indian River Lagoon (IRL) was the site of an unknown manatee mortality episode following a prolonged non toxin producing brown tide (*Aureoumbra lagunensis*) algal bloom. While the brown tide was not implicated as the direct cause of the manatee deaths, it was associated with the massive depletion of seagrass, a food staple for manatees. An estimated 60% loss in the region’s seagrass beds was reported after the brown tide algal mats blocked the penetration of the sunlight needed to sustain the seagrass populations in the water column^2,3^. One hypothesis for the mortality episode in the Indian River Lagoon was that the deaths were attributable to the manatee’s ingestion of alternate food sources, including red drift algae (*Gracilaria*), following the loss of their typical food source. While manatee ingestion of *Gracilaria* was documented before in 1984, it is a non-typical food source for them^4^.

Of the 830 deaths reported statewide in 2013 from all causes, 152 in Brevard County (Indian River Lagoon (IRL)) were associated with an undetermined cause and 277 were associated with a red tide in Southwest, Florida^4^. Of the red tide associated deaths, the majority were reported in the Fort Myers/SW Florida area with 86% of those deaths occurring in Lee County-the epicenter of a large algal bloom of *Karenia brevis* (‘red tide’) near the mouth of the Caloosahatchee River^1^. The bloom began in late 2012 and persisted to March 2013, causing brevetoxin (PbTx) to be emitted into the surrounding waters for a prolonged period^1^. Concentrations of PbTX can be detected in blood in affected marine mammals at low levels in the range of 3–16 ng/ml as seen in dolphins after a large mortality event associated with red tide^5^. In manatees that succumbed at the time, PbTX varied by organ, with highest concentrations found in their stomachs (range 61 to 1132 ng PbTX/g) followed by the liver (58 to 300 ng PbTx/g)^5^.

To investigate the mortality episodes in 2013, two proteomic experiments were conducted on serum collected from live manatees sampled either in the IRL at the time of the die off or in manatees recovering from red tide at Zoo Tampa. These samples were compared to serum samples from healthy manatees sampled during routine health assessments at Crystal River. The goal of the project was to explore the differences in serum protein concentrations and identify potential protein biomarkers in manatees from populations impacted by mortality episodes such as those seen in a diagnosed biotoxin event, brevetoxicosis, and a potential unknown toxin involved in the IRL mortalities. Performing a global analysis of the serum proteome of impacted manatees compared to controls provided potential novel biomarkers associated with the stressors. Protein biomarkers can be used to develop diagnostic assays for early stages of disease and may also be useful to understand biochemical pathways that contribute to morbidity.

## Results

### 2D-DIGE analysis

**Figure 1:**
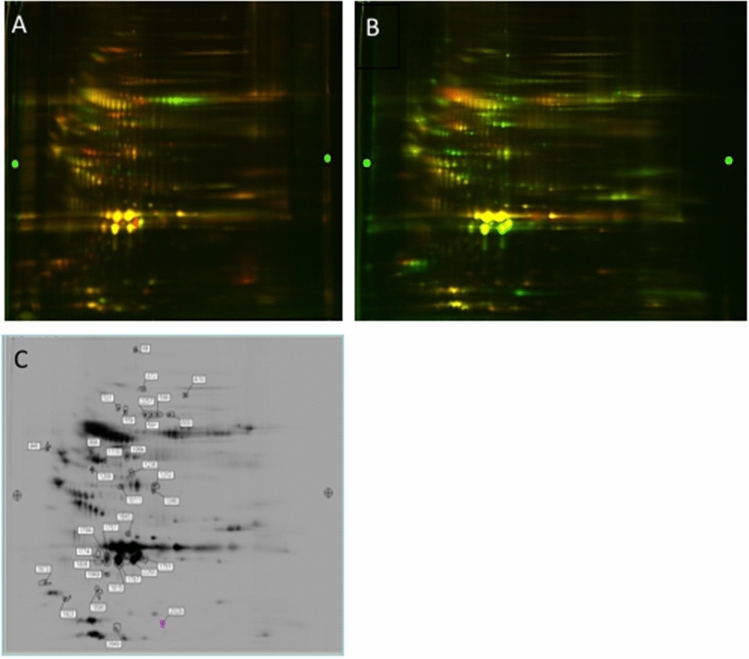
Differential in gel electrophoresis (DIGE) separation of serum proteins. After labeling, the extracted proteins from the serum samples were mixed and co-electrophoresed through the first- and second-dimension gels. **(A)** Comparison of a serum protein sample from a red tide manatee labeled with Cy3 (green dye) with a serum protein sample from a Crystal River manatee labeled with Cy5 (red dye). **(B)** Comparison of a serum protein sample from an IRL manatee labeled with Cy5 (red dye) with a serum protein sample from a Crystal River manatee labeled with Cy3 (green dye). **(C)** Coomassie blue stained gel showing the positions of the spots that were identified as differentially expressed for the gel in **(A)** and cut out for identification by mass spectrometry.

Selected 2D-DIGE gels are shown that illustrate comparisons of samples from a manatee from the red tide group (Fig. 1A, green) with a control sample from a Crystal River manatee (Fig. 1A, red) and a sample from the IRL group (Fig. 1B, red) with a sample from a different Crystal River manatee (Fig. 1B, green). A Coomassie blue stained gel showing the positions of the spots that were cut for protein identification for the gel in Fig. 1A is shown in Fig. 1C. In total, 2,271 spots were detected. Only a subset of spots with a volume average ratio above or below a 1.5-fold change (p-value < 0.05) were selected for further analysis. Proteins detected in the samples from the red tide or the IRL groups, which showed increased or decreased abundances in comparison to controls were identified in 26 spots (Supplementary Table 1). Proteins that were also detected by the iTRAQ method are labeled with an asterisk. The differentially expressed proteins found in the highest number of spots, excluding spots identified as keratin, were C-reactive protein (n = 10 spots), complement C4-A (6 spots), complement C3 (4 spots), gelsolin (4 spots), and serum albumin (4 spots), suggesting that these proteins may be present in multiple isoforms with different post-translational modifications.

2D-DIGE showed 17 differentially expressed proteins in the red tide group and 9 differentially expressed proteins in the IRL group compared to Crystal River manatees. The proteins ceruloplasmin, complement C4-A, and vitronectin were differentially increased in both the red tide and IRL groups.

### iTRAQ results

There were 263 proteins identified for the red tide group and 311 for the IRL group that were differentially expressed but only 60 proteins in the red tide group and 48 proteins in the IRL group were considered significantly differentially expressed (p-value < 0.05). Proteins that were also detected by the 2D-DIGE method are listed with an asterisk (Supplementary Tables 2 and 3).

### Red tide group proteins (n = 60)

Of the 60 proteins identified for the red tide group (Supplementary Table 2), 34 passed the fold change selection criteria, with 16 above a 1.2-fold change and 18 below a 0.8-fold change. The proteins with average fold change (FC) above 1.2-fold were complement C3 (FC 1.42), complement C4-A (FC 1.83), vitronectin (FC 1.57), ceruloplasmin-like protein (FC 2.32), C-reactive-like protein (FC 1.68), angiotensinogen (FC 2.08), gelsolin (FC 1.41), thrombospondin-1 (FC 1.36), heparin cofactor 2 (FC 1.50), sulfhydryl oxidase 1 (FC 1.65), complement C1s subcomponent (FC 1.33), fibulin-1 isoform 2 (FC 1.24), pyruvate kinase isozymes M1/M2 (FC 2.29), *N*-acetylmuramoyl-l-alanine amidase (FC 1.48), and complement C1q subcomponent subunit B (FC 1.46). Functions of these proteins are listed in Table 1.

**Table 1:** Functions of a select group of increased proteins identified in the red tide group.

Increased proteins^a^	Function (obtained from UniProt and NCBI)
Complement C3	Effector of the innate and acquired immune system, activator of the complement system, and local inflammatory mediator
Complement C4-A	Complement activation and inflammatory response activation
Vitronectin	Cell adhesion and cellular defense
Ceruloplasmin	A copper-binding glycoprotein that assists with iron transport
C-reactive protein	Host defense, promotes agglutination and phagocytosis, scavenger of nuclear material released from damaged circulating cells, increases during infection, injury, or inflammation
Angiotensinogen	Main component in renin-angiotensin system, regulates blood pressure
Gelsolin	Binds to actin to cap it and block exchange and has a role in ciliogenesis. Its accumulation is related to generalized amyloidosis
Thrombospondin-1	Glycoprotein involved in adhesion, mediates cellular matrix interactions, binds heparin, partly responsible for dental pulp maintenance, part of ER stress response, ligand for agents with antiangiogenic properties
Heparin cofactor 2	Inhibitor of thrombin and also chymotrypsin, has chemotactic ability for monocytes/neutrophils
Sulfhydryl oxidase 1	Catalysis of sulfhydryl groups oxidation and disulfide bond formation, may have tumor-suppressing capabilities in fibroblasts
Complement C1s subcomponent	First component in the complement cascade. It has collagen-like regions that interact with Ca^2+^-dependent proenzymes, activation is part of IgG and IgM immune response
Fibulin-1 isoform 2	Found in the extracellular matrix, performs cellular adhesion, possible role in tumor suppression, and helps maintain hemostasis. Modulator of amyloid precursor protein
Pyruvate kinase isozymes M1/M2, 3	Regulate glycolysis
*N*-acetylmuramoyl-l-alanine amidase	Scavenger of peptidoglycan (PGN) in bacterial cell walls
Complement C1s sub-component subunit B	Associated with a complex that yields C1 complement, has collagen-like regions that interact with Ca^2+^-dependent proenzymes

^a^Proteins were selected that had average fold change ratios > 1.2.

The proteins with average fold changes at or below 0.8 were prothrombin (FC 0.8), C4b-binding protein beta chain (FC 0.78), C4b-binding protein alpha chain (FC 0.77), CD5 antigen-like (FC 0.76), vitamin D-binding protein (FC 0.75), inter-alpha-trypsin inhibitor heavy chain H2 (FC 0.71), coagulation factor XIII A chain (FC 0.7), immunoglobulin J chain (FC 0.7), insulin-like growth factor-binding protein complex acid labile subunit (FC 0.7), antithrombin-III isoform 1 (FC 0.69), lumican (FC 0.69), inter-alpha-trypsin inhibitor heavy chain H3 (FC 0.67), alpha-1B-glycoprotein (FC 0.65), fibrinogen alpha chain (FC 0.62), fibronectin isoform 3 (FC 0.57), inter-alpha-trypsin inhibitor heavy chain H1 (FC 0.57), histidine-rich glycoprotein (FC 0.56), kininogen-1 isoform 1 (FC 0.54), and transthyretin (FC 0.54).

### IRL group proteins (n = 48)

Of the 48 proteins identified for the IRL group (Supplementary Table 3), 10 passed the fold change selection criteria, with 9 above a 1.2-fold change and 1 below a 0.8-fold change. The increased proteins with significant average fold change ratios above 1.2-fold in the IRL group were complement C4-A (FC 1.25), vitronectin (FC 1.25), histidine-rich glycoprotein (FC 1.34), kininogen-1 isoform 1 (FC 1.38), ceruloplasmin-like protein (FC 1.22), inter-alpha-trypsin inhibitor heavy chain H2 (FC 1.21), alpha-2-HS-glycoprotein (FC 1.22), properdin (FC 1.30), and alpha-1-microglobulin/bikunen precursor (AMBP) (FC 1.38). The only decreased protein with a fold change below 0.8 was transthyretin (FC 0.7). Functions of the upregulated proteins are found in Table 2.

**Table 2:** Functions of a select group of increased proteins identified in the IRL group.

Increased proteins^a^	Function (obtained from UniProt and NCBI)
Alpha-2-HS-glycoprotein	Has a functional role as an endocytosis promoter. Found in mineral bone matrix, has opsonic properties, and shows affinity to barium and calcium
Ceruloplasmin	A copper-binding glycoprotein that assists with iron transport
Complement C4-A isoform 1	Activation of the complement cascade and inflammatory response
Histidine-rich glycoprotein	Pathogen clearance, angiogenesis, fibrinolysis, coagulation, and regulates immune function. Adapter protein that performs necrotic cell clearance by promoting phagocytosis
Inter-alpha-trypsin inhibitor heavy chain h2	Forms complexes with and/or carries hyaluronan. This complex has a role in inflammatory response
Kininogen-1 isoform 1	Inhibits thiol proteases and assists with preventing blood coagulation and clotting and certain subunits have a cardio-protective function
Properdin	Regulates the alternate complement pathway and binds to C3 and C5 convertase enzyme complexes
Protein AMBP	Trypsin inhibitor. It also inhibits lysosomal granulocytic elastase and plasmin and is a calcium oxalate crystallization inhibitor
Vitronectin	Cell adhesion and cellular defense

^a^Proteins were selected that had average ratios > 1.2.

### Western blot results

**Figure 2:**
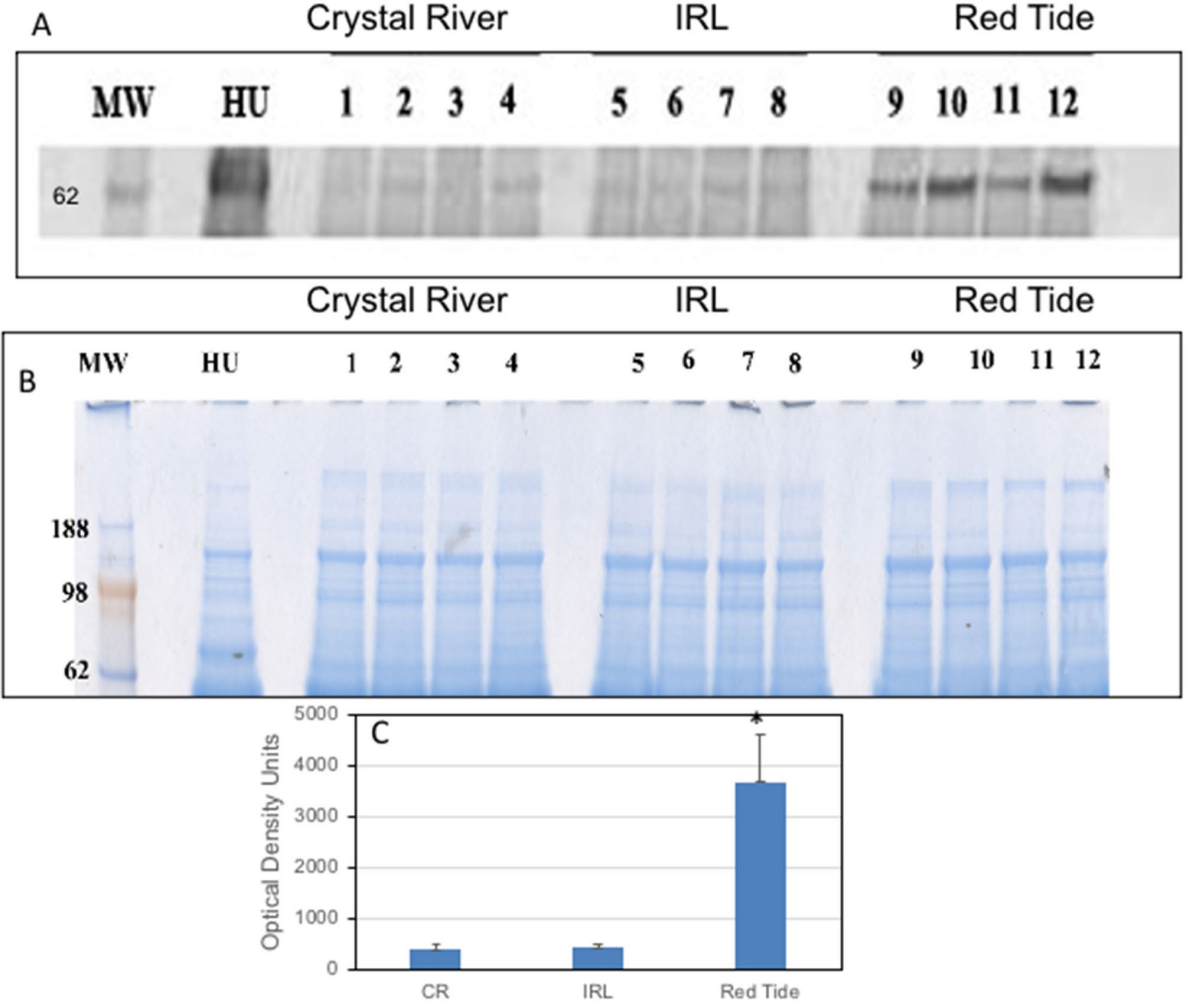
Confirmation of up regulation of Protein C4-A in the serum of manatees from the red tide group. **(A)** Western blot of protein electro transferred to a PVDF membrane and probed with rabbit anti-human protein C4 antibody [N1N2-2]. **(B)** Corresponding Coomassie blue stained gel showing equal loading of proteins in the wells. **(C)** ImageJ quantification of bands in the Western blot. *Statistical significance at p-value < 0.05. The original gel and blot are shown if supplementary Fig. 2.

Biomarkers for further verification by Western blot were chosen based on their statistically significant increase or decrease in the 2-DIGE and iTRAQ experiments and if there were commercially available antibodies for mammalian homologs. The homologies for each protein were as follows: ceruloplasmin (CP) (88% humans, 83% rabbit), C4-A (84% human, 82% rabbit), transthyretin (TTR) (human 82%, 84% rabbit), and kininogen (KNG1) (human 70%, 72% rabbit). Of the antibodies chosen, only one against human C4-A worked by Western blot, suggesting that the epitopes were not conserved across species. Human C4 is composed of three subunits of MW 95, 75 and 33 KDa. It is likely that manatee C4 is also composed of three subunits. The antibody, however, only recognized the 75 KDa subunit (Supplementary Fig. 2). Thus, C4-A was the only protein validated through the Western blot experiment (Fig. 2). Densitometry of the increase in C4-A protein showed a significant 9.8-fold increase over the concentration present in serum of Crystal River manatees. There was a 15% increase in this protein for the IRL manatees, but this was not significantly different from controls.

## Discussion

This proteomic survey was conducted to identify proteins that were differentially expressed in the serum of manatees affected by two distinct mortality episodes: a red tide group and an unknown mortality episode group in the IRL. These groups were compared to a control group sampled at Crystal River. The red tide group’s exposure was evidenced by the presence of the PbTx antigen, with brevetoxin values in the 4.3 to 14.4 ng/ml range. The other group did not present with clinical symptoms except for mild cold stress in some animals. Two proteomics approaches were employed, 2D-DIGE and shot gun proteomics using LC–MS/MS, which provided similar results, suggesting that several serum proteins were specifically altered in each of the manatee mortality episode groups compared to the Crystal River control group. The differentially expressed serum proteins were cautiously identified based on annotation of the manatee genome^6,7^ and their amino acid sequence homologies with human serum proteins. While additional work still needs to be done to confirm that the identified manatee proteins function similarly to their human homologs, possible insight on the function of the proteins can be derived from human studies.

The two proteomics methods used, 2D-DIGE and iTRAQ LC–MS/MS are complementary and both rely on LC–MS/MS for protein identification. 2D-DIGE is a top-down approach, quantifying the differentially expressed proteins at the protein level before identifying the protein by LC–MS/MS, while the iTRAQ method is a bottom-up approach, where the whole proteome is first digested with trypsin, the generated peptides are separated by chromatography and identified and measured by mass spectrometry. Mass spectrometry has become the primary method to analyze proteomes, benefitting from genomic sequences and bioinformatics tools that can translate the sequences into predicted proteins. There are excellent reviews of proteomics methods and how they may be used across species^8,9^.

In total, 19 of the 26 proteins identified using the 2D-DIGE method were also identified by iTRAQ (Supplementary Table 1) which showed that these findings were replicated using two complementary experimental methods. In the 2D-DIGE method, most of the proteins were found in multiple spots, suggesting that they were differentially modified. 2D-DIGE can separate proteins based on a single charge difference. Some of the spots contained multiple proteins so it was difficult to determine the fold change of each of the proteins in these spots. For example, protein C4A was identified in 7 different spots, likely representing multiple isoforms. We were not able to corroborate the different post-translational modifications (PTMs) with iTRAQ, as the experiment was not designed to look for PTMs, only total protein quantitation. A drawback of 2D-DIGE is that keratin introduced into the sample from reagents at the time of electrophoresis or through the multiple steps required for protein extraction is also seen in the gels^10–12^. It is unlikely that the keratins were from the serum samples, as blood was collected directly into vacuum tubes. Because of the issue of keratin contamination, the 2D-DIGE method is considered more qualitative in its determination and thus in this study, iTRAQ data were the primary basis for quantitation.

Pathway analysis detects groups of proteins that are linked in pathways that may be related to disease processes. We used Pathway Studio using subnetwork enrichment analysis to determine disease pathways potentially in place for the red tide and IRL manatees. The Pathway Studio database is constructed from relationships detected between proteins and diseases from articles present in Pubmed but is heavily directed towards human and rodent proteomes. To be able to use this tool, we assigned human homologs to the identified manatee proteins, assuming that based on their sequence homology the proteins would function in a similar way. There are many studies that suggest this assumption has merit, for example Nonaka and Kimura have examined the evolution of the complement system and found clear indications of homology among vertebrates^13^.

The top 20 pathways for the red tide group (Table 3) and the IRL group (Table 4) show the diverse set of molecular pathways that may be affected by the exposures. Many of the same pathways appeared for both groups including thrombophilia, inflammation, wounds and injuries, acute phase reaction and amyloidosis. Thrombophilia was the most upregulated pathway for the IRL group (p-value 1.10E-19) and the second most upregulated pathway for the red tide group (p-value 4.1E-19). Thrombophilia, a condition in which blood clots occur in the absence of injury, happens when clotting factors become unbalanced. We obtained proteomics information on 12 of the proteins in this pathway, with some moving in opposing directions. The dysregulated proteins that were increased for both red tide and the IRL groups were SERPIN D1 (Serpin family member D 1), CRP (C-reactive protein), and PLAT (plasminogen activator) and the ones that were decreased in both groups, were SERPIN C1 (Serpin family member C 1), F5 (coagulation factor 5), and ALB (albumin). One protein, AGT (angiotensinogen), was upregulated in the red tide group but downregulated in the IRL. HRG (histidine rich glycoprotein), PROS1 (Protein S), C4BPA (complement component 4 binding protein alpha, and F2 (coagulation factor 2, also known as prothrombin) were downregulated in the red tide group but upregulated in the IRL group. The disparate regulation of proteins in this pathway suggests that clotting was among the pathways disrupted in the affected manatees. Red tide exposed manatees often present with hemorrhagic issues in their intestines, lungs and the brain (^14^), suggesting that downregulation of coagulation factors may be responsible for this clinical evaluation. Interestingly HRG was upregulated in the IRL by 1.34-fold and downregulated in the red tide group by 0.56-fold, making this protein a good biomarker to distinguish the two events.

**Table 3:** Subnetwork enrichment pathways for serum proteins obtained from manatees exposed to red tide.

Rank	Gene set seed	^a^Total # of neighbors	^b^Overlap	^c^Percent overlap	^d^p-value
1	Atherosclerosis	983	27	2	7.81E−22
2	Thrombophilia	68	12	17	4.10E−19
3	Inflammation	1920	31	1	6.08E−19
4	Myocardial Infarction	517	20	3	1.71E−18
5	Thrombosis	442	19	4	2.25E−18
6	Wounds and Injuries	1729	29	1	8.06E−18
7	Sepsis	450	16	3	3.92E−14
8	Venous thromboembolism	20	7	33	1.01E−13
9	Acute-phase reaction	185	11	5	2.96E−12
10	Fibrosis	733	17	2	5.10E−12
11	Lupus erythematosus, systemic	345	13	3	7.47E−12
12	Coronary artery disease	210	11	5	1.17E−11
13	Dissem. intravascular coagulation	68	8	11	1.57E−11
14	Cardiovascular diseases	494	14	2	4.49E−11
15	Cardiovascular passage	126	9	7	6.87E−11
16	Proteinuria	325	12	3	6.96E−11
17	Neutrophil accumulation	187	10	5	9.29E−11
18	Amyloidosis	90	8	8	1.52E−10
19	Stroke	468	13	2	3.28E−10
20	Diabetic retinopathy	155	9	5	4.36E−10

^a^The number of proteins in the specified pathway for human proteins.

^b^The number of proteins from the data set that matched proteins in the pathway.

^c^The calculated percent of proteins in the data set divided by the proteins in the human set times 100.

^d^The probability that the pathway is enriched.

**Table 4:** Subnetwork enrichment pathways for serum proteins obtained from manatees sampled in the IRL.

Rank	Gene set seed	^a^Total # of neighbors	^b^Overlap	^c^Percent overlap	^d^p-value
1	Thrombophilia	68	12	17	1.10E−19
2	Wounds and injuries	1729	29	1	1.27E−19
3	Atherosclerosis	983	24	2	3.21E−19
4	Inflammation	1920	29	1	2.34E−18
5	Thrombosis	442	18	4	7.15E−18
6	Myocardial infarction	517	16	3	5.65E−14
7	Sepsis	450	15	3	1.46E−13
8	Hemorrhage	432	14	3	1.68E−12
9	Dissem. intravascular coagulation	68	8	11	6.89E−12
10	Cardiovascular diseases	494	14	2	1.02E−11
11	Venous thromboembolism	20	6	28	1.04E−11
12	Cardiovascular passage	126	9	7	2.73E−11
13	Amyloidosis	90	8	8	6.73E−11
14	Stroke	468	13	2	8.49E−11
15	Fibrosis	733	15	2	1.53E−10
16	AcutE−phase reaction	185	9	4	8.34E−10
17	Neutrophil accumulation	187	9	4	9.16E−10
18	Blood coagulation disorders	45	6	13	1.72E−09
19	Coronary artery disease	210	9	4	2.54E−09
20	Proteinuria	325	10	3	7.06E−09

^a^The number of proteins in the specified pathway for human proteins.

^b^The number of proteins from the data set that matched proteins in the pathway.

^c^The calculated percent of proteins in the data set divided by the proteins in the human set times 100.

^d^The probability that the pathway is enriched.

**Figure 3:**
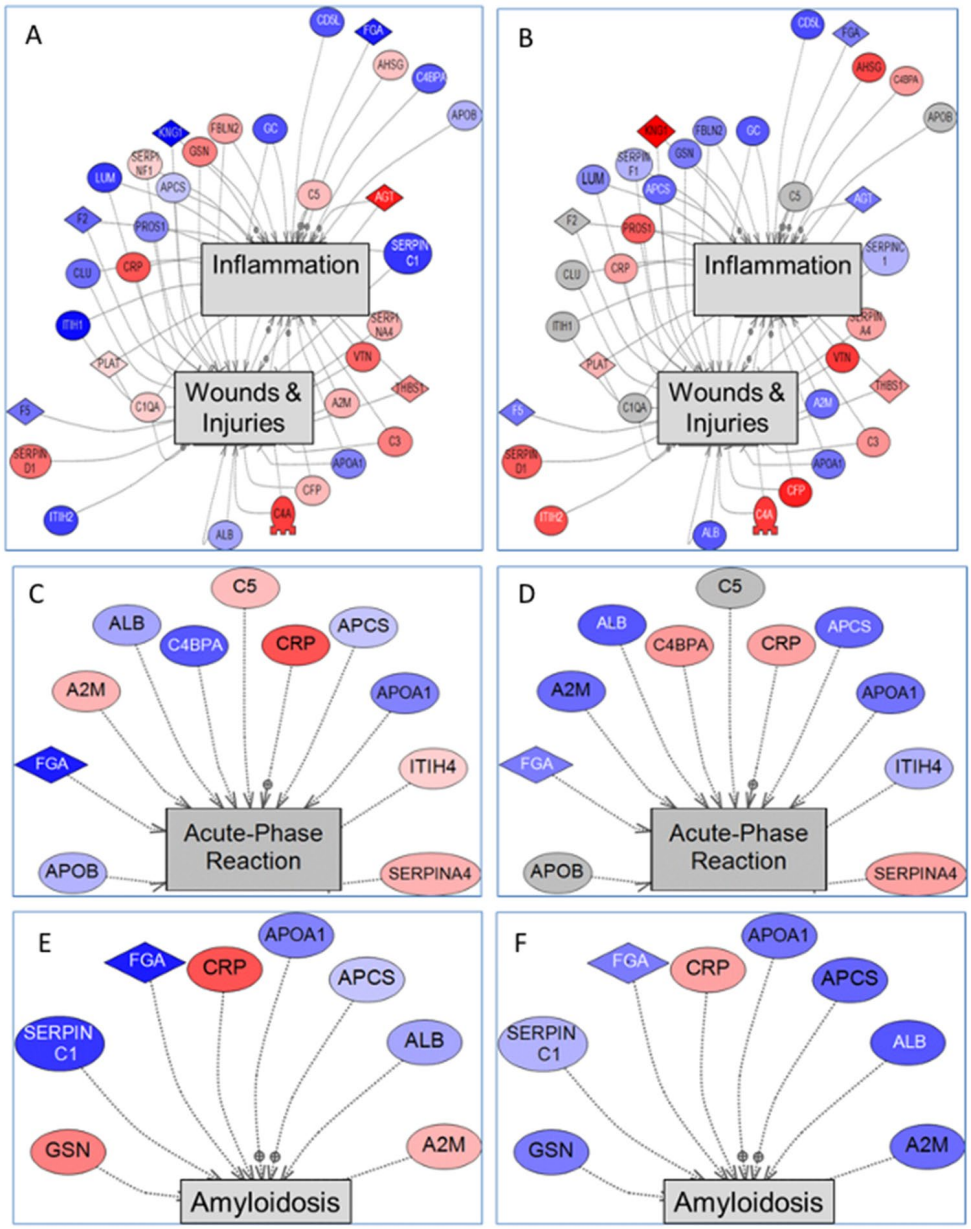
Pathway Studio images of subnetwork enrichment analysis (SNEA) of **(A,B)** the combination of inflammation and wounds and injuries, **(C,D)** acute phase reaction pathway and **(E,F)** amyloidosis pathway. The proteins that were identified by iTRAQ for the two groups (red tide and IRL) were compared to the Crystal River control group by Pathway Studio using subnetwork enrichment analysis. **(A,C,E)** Pathways built with differentially regulated proteins from serum of manatees exposed to red tide; **(B,D,F)** Overlay of differentially regulated proteins from serum of manatees collected from the IRL on the same pathways. Red, increased protein amount; blue, decreased protein amount, and gray, protein is not altered.

Among the manatees in the red tide group, inflammation was ranked 3rd (p-value < 6 E−19) and wounds and injuries, 6th (p-value < 8E−18), while for the IRL group, wounds and injuries was ranked 2nd (p-value < 1.3 E−19) and inflammation, 4th (p-value < 2 E−18). Given the importance of these pathways related to acute immunological reaction, their graphical interpretations (including their component entities) are shown in Fig. 3. There is significant overlap in proteins that are associated with inflammation and wounds and injuries in the two groups, Fig. 3 A,B. The combined pathways were built first on the data that were obtained from the red tide group (Fig. 3A). Using the same set of entities, we overlaid the data from the IRL group (Fig. 3B). In these pathways, red indicates proteins that are increased, while blue indicates proteins that are decreased, and gray denotes proteins that were unchanged. The intensity of the color is proportional to the degree of increase or decrease. While many of the proteins are altered in the same direction in both the red tide and IRL groups, some of the proteins are altered in opposite directions, for example ITIH2 (inter-alpha (globulin) inhibitor H2), GSN (gelsolin), and KNG1 (kininogen), among others, suggesting that the exposures were quite distinct. Interestingly, red tide exposure has been correlated to immune dysfunction in manatees, since isolated lymphocytes from manatees exposed to red tide are less able to proliferate when exposed to a mitogen such as Concanavalin A (ConA) or phytohemagglutinin (PHA)^15^.

Proteins that comprise the acute phase reaction pathway showed increased concentrations in both the red tide and IRL manatees (Fig. 3C,D). The pathway was built on the data obtained from the red tide group (Fig. 3C) and overlaid with the IRL group (Fig. 3D). This pathway includes the complement cascade, which is an integral part of innate immunity^16^, which is highly conserved among vertebrates^13^. Comparisons to the well-studied system in humans provides hypotheses as to what might be happening in manatees. The complement pathway in mammals is comprised of over 50 different proteins that act together to facilitate inflammation and immune response, promote phagocytosis, promote antibody/antigen binding, and attack the membrane of foreign invaders^17^. The complement cascade is most often activated by antibody-antigen binding which instigates the activation of complement 3 (C3)^18^. The most common initiating event is C1q, which binds to several molecules such as C-reactive protein and undergoes a conformation change^16^. Both C3 and C4 and their molecular subcomponents are early actors in the complement cascade that activate later proteins in the pathway, such as C5-C9 activation, which can cause self-inflicted damage to the cell or attack foreign microbial agents^16^.

In the red tide manatees, the complement cascade protein complement C1s subcomponent (FC 1.33), complement C1q subcomponent subunit B (FC 1.46), complement C3 (FC 1.42), and complement C4-A isoform 1 (FC 1.83) were all upregulated. Western blot, used as an orthogonal validation of C4-A upregulation in the red tide group, showed it to be increased by 9.8-fold. iTRAQ is known to underestimate the actual increase in proteins due to suppression from the reactive chemistry of the isobaric tags^19^. Since the rabbit anti-human C4-A antibody cross reacts with the manatee homolog, this could be used to create an assay. C4 is not very specific for red tide, as other insults may change its expression. However, it may be useful if included in a panel of blood proteins.

In the IRL manatees, the C4-A (FC 1.25) showed a small increase in expression over controls in the iTRAQ experiment and the increase was confirmed by the Western blot, however the change was not significant compared to controls. Other members of the complement cascade, which also appeared among the proteins, were at most increased by only 10% over controls and these also were not significant. Thus, the complement cascade may not represent a major pathway for IRL manatees.

Interestingly, while many of the same entities were altered in the Acute Phase Reaction Pathway, the pattern of alteration was different for the two groups. Some of the entities were regulated in the same direction but some in reverse direction. Two examples for regulation in reverse direction are alpha-2-macroglobulin (A2M) and complement component 4 binding protein (C4BPA), where A2M was upregulated in manatees exposed to red tide and downregulated in manatees from the IRL and C4BPA was downregulated in the red tide group but upregulated in the IRL group.

The amyloidosis pathway is also important to consider for manatees exposed to red tide and the IRL mortality episode (Fig. 3E,F). Previous studies have shown a relationship between serum amyloid protein (SAA) with an increased prevalence of inflammation^20,21^. SAA is an acute phase protein and is one of the few existing biomarkers used to diagnose pre-existing illness and inflammation in manatees. It is routinely measured during manatee health assessments^21,22^ and thus for the current study, we have SAA values for the manatees sampled during health assessment in Brevard County and Crystal River. This protein was not measured for the red tide manatees that were recovering at Zoo Florida. All of the SAA values were in the normal range (< 50 mg/ml)^21^, except for one manatee from Crystal River, which was slightly out of range at 60 mg/ml. SAA values > 80 mg/ml to 1200 mg/ml are considered truly elevated and are likely to be present in disease states^21^. SAA was not identified in either the 2D-DIGE or iTRAQ experiments in this study. Instead, other acute phase proteins were evaluated. These included C-reactive protein (CRP), which was highly upregulated in the red tide group (Fig. 3E) and slightly upregulated in the IRL group (Fig. 3F), and serum amyloid P-component (APCS), which was downregulated in the IRL group (AR 0.81) (Fig. 3F) and to a lesser extent in the red tide group (AR 0.96) (Fig. 3E). CRP has not been previously evaluated in manatees because available commercial mammalian antibodies do not apparently cross react^21^.

In the current study, other proteins classified as regulators of amyloidosis^23^ were also altered including albumin (ALB)^23^, alpha-2-macroglobulin (A2M)^24^, apolipoprotein A1 (APOA1)^25^, fibrinogen alpha chain (FGA)^26^, gelsolin (GSN)^27,28^, and serpin peptidase inhibitor C1 (SERPINC1)^29^, among others. Amyloidosis occurs when an abnormally high amount of amyloid protein begins to accumulate and form fibrils and deposits, which can disrupt normal tissue architecture^30,31^. Fibrils are bundles of misfolded proteins that form deposits^32^. In Alzheimer's disease, the amyloid β precursor protein (APP) accumulates and forms a plaque in the brain^33^.

The amyloid protein transthyretin^34^ was decreased in the red tide (FC 0.54) and IRL (FC 0.70) groups. Transthyretin transports thyroxine (T4) and triiodothyronine (T3) thyroid hormone and retinol so a decrease in expression may result in decreased thyroid function or an increase in amyloidosis^35^. Two other proteins of note were vibronectin and gelsolin. Vibronectin is a component of the extracellular matrix in higher animals^36^ and it has been associated with amyloid plaques in Alzheimer’s disease^37^. Gelsolin regulates actin binding in a calcium dependent process and has been found in blood vessels and tissue basement membranes of patients with wide-spread systemic amyloidosis^27,31^. Both of these proteins were over expressed in the red tide group (vitronectin, FC 1.57: and gelsolin, FC 1.41). The IRL manatees only had increased expression of vitronectin (FC 1.25).

Clearly the manatees that were confirmed to have been exposed to red tide, exhibited by the brevetoxin values in their blood, were sicker than the manatee samples collected in the IRL. The intensity of expression of the differentially expressed proteins support this observation. Some of the manatees sampled in the IRL had clinical symptoms of mild cold exposure but were also in the vicinity of the unknown mortality episode in the IRL and they may have had subclinical effects. It is likely that the changes in the serum proteome reflected both of these stressors.

This proteomic analysis identified additional serum proteins that may serve as biomarkers of disease after further evaluation and validation. The proteins with the top five-fold changes for the red tide group were ceruloplasmin-like (FC 2.32), pyruvate kinase isozymes M1/M2 (FC 2.29), angiotensinogen (FC 2.08), complement C4-A (FC 1.83), and C-reactive protein (CRP) (FC 1.68). Ceruloplasmin’s main function is in iron and copper homeostasis and it is increased when iron levels in the brain are elevated due to oxidative stress^38^. For the IRL group, the proteins with the top five highest average fold change included kininogen-1 isoform 1 (FC 1.38), protein AMBP (FC 1.38), histidine-rich glycoprotein (FC 1.34), properdin (FC 1.30), and complement C4-A (FC 1.25).

The most under expressed proteins in the red tide manatee group included transthyretin (FC 0.54), kininogen-1 isoform 1 (FC 0.54), histidine-rich glycoprotein (FC 0.56), inter-alpha-trypsin inhibitor heavy chain H1 (FC 0.57), and fibronectin isoform 3 (FC 0.57). In the IRL group, transthyretin (FC 0.70) was the most decreased protein. While many of the proteins identified through this survey serve various cellular functions, several key biological roles including inflammatory and immune response, the complement cascade activity, acute-phase response, amyloid accumulation, and iron and copper homeostasis, were increased in the samples. Ceruloplasmin (CP) was the most increased protein in the red tide group and its association with oxidative stress and neurodegenerative effects may reflect direct effects of red tide in the brains of affected manatees^38,39^, which includes disorientation, seizing and inability to surface, resulting in drowning^14^.

Manatees sampled in the IRL, during the unknown mortality event, also presented with elevated levels in specific serum proteins, which may point to a compromised immune system. Since many undiagnosed manatee mortalities occur every year, developing enhanced diagnosis criteria in the form of biomarkers will help researchers improve their diagnosis and treatment protocols. Proteins related to amyloid formation and the complement cascade may serve as potential markers of disease after careful validation. We recommend that these potential biomarkers receive further validation with a larger group of manatees. CRP was previously a candidate for evaluation for manatees, but in the absence of a commercial antibody was abandoned^21^. With manatee-specific antibodies, it might be possible to develop specific assays for each of these proteins.

## Materials and methods

### Study design

Two complementary proteomic experiments using either two-dimensional difference gel electrophoresis (2D-DIGE) or isobaric tags for relative and absolute quantification (iTRAQ) were conducted to determine the variations in serum protein abundance of two manatee populations compared to controls from Crystal River. 2D-DIGE separated proteins in gels and quantitation was carried out by measuring the intensity of protein in each stained spot, while iTRAQ used a shotgun approach by directly separating peptides from trypsin digested proteins by chromatography and quantifying differences at the level of mass spectrometry. Twelve serum samples from three groups (red tide (n = 4), IRL (n = 4), and controls (n = 4)) were included in the study (Table 5). Supplementary Fig. 1 shows how the iTRAQ labels were used to label the 12 samples and depicts the overall study design that was used for both the 2D-DIGE and 8-plex iTRAQ experiments.

**Table 5:** Characterization of manatees from which serum samples were drawn.

ID	^a^Group	Date of collection	Gender	^b^Age	^b^Weight (lbs)	^b^Length (cm)	^c^Clinical notes
CCR1301	Control	2/12/2013	F	Adult	1066	288	Parent in a mother–child pair
CCR1303	Control	2/12/2013	M	Adult	858	269	Good blood chemistries
CCR1305	Control	2/12/2013	M	Adult	1248	295	High creatine kinase
CCR1307	Control	2/13/2013	M	Sub-adult	584	229	High serum amyloid A (SAA)
CBC 1201	IRL	12/14/2012	M	Adult	790	260	Mild cold exposure
CBC1205	IRL	12/14/2012	M	Adult	812	262	Mild cold exposure
CBC1206	IRL	12/14/2012	M	Adult	874	261	Mild cold exposure
CBC1211	IRL	12/15/2012	M	Sub-adult	636	249	Signs of cold exposure
T13-0833	Red tide	1/17/2013	M	–	–	–	PbTx-3 (ng/mL) 10.8
T13-0841	Red tide	2/21/2013	M	–	–	–	PbTx-3 (ng/mL) 4.3
T13-0844	Red tide	2/28/2013	M	–	–	–	PbTx-3 (ng/mL) 14.4
T13-0851	Red tide	3/5/2013	M	–	–	–	PbTx-3 (ng/mL) 6.1

^a^Group, controls, collected from Crystal River manatees during annual health assessments, IRL, manatees collected from the Indian River Lagoon, Red tide, manatees recovering from red tide at Zoo Florida.

^b^Data on age, weight and length were not available for manatees that recovered at Zoo Florida.

^c^PbTx-3 is the brevetoxin produced during red tide bloom.

All methods were carried out in accordance with relevant guidelines and regulations. This project was approved by the University of Florida Institutional Animal Care and Use Committee (IACUC), U.S. Geological Survey Sirenia Project (USGS permit #: MA791721-5) and Florida Fish and Wildlife Conservation Commission (FWC) (FWC permit #: MA067116-1). The study complied with the ARRIVE guidelines^40^. The manatees employed for this study were field captured and as such were randomly selected for each of the groups. There were only 4 manatees affected by red tide and all 4 were selected. The proteomics experiment plan was developed prior to the analysis of the samples and strictly followed proteomics guidelines for analysis including statistical analysis, but the plan was not preregistered. The manatees were grouped by location, but the identities of the groups were blinded to the technicians performing the analysis. The raw proteomics iTRAQ data has been deposited at PRIDE and is accessible.

The red tide group was comprised of manatees treated at Zoo Tampa (Florida) for red tide-related symptoms. The IRL group was comprised of animals from the IRL, which was the site of the 2013 East coast manatee mortality episode and these manatees also presented with signs of mild cold stress. While the IRL group did not present with acute illness, they were sampled in an area where many manatees were dying, and presumably they were exposed to the same environmental factors. The control group was comprised of manatees from Crystal River, Florida that presumably were not known to have been influenced by the mortality episodes. The manatees from the IRL and control groups were screened during routine annual manatee health assessments of free-ranging manatees conducted by USGS and FWC. To obtain serum, blood samples collected from sub adult and adult manatees without anticoagulant, were centrifuged within 1 h of collection and serum was transported on dry ice and stored later at -80 °C. Samples from Zoo Tampa were shipped overnight on dry ice and stored -80 °C. Approximately 1–2 mL of serum was transferred to the Interdisciplinary Center for Biotechnology Research (ICBR) at the University of Florida for inclusion in this study.

### Proteomics experiments

Total protein was extracted from the serum samples and quantified with the EZQ protein quantitation kit (Invitrogen, CA), according to manufacture instructions and ranged between 70 and 89 mg/mL. Equal concentrations of protein (10 mg) for each of the samples was further treated with ProteoMiner columns (BioRad Laboratories, Hercules, CA), following best practices for serum proteomics^41^. The enriched total protein from each sample was used for 2D-DIGE and iTRAQ experiments. We required a total of 600 µg of each protein sample for 2D-DIGE and 100 µg from each sample for iTRAQ. An additional pooled sample was made as a reference sample for 2D-DIGE, containing a mixture of 50 µg of each sample.

### 2D-DIGE

The protein samples were solubilized in 2D-lysis buffer containing 4% (w/v) CHAPS, 7 M urea, 30 mM Tris, and 2 M thiourea and labeled with Cy3 or Cy5 (CyDye DIGE Fluor kit), using a minimal dye approach. Samples were randomized for labeling with the dyes in order to minimize batch effects from labeling, if any should occur. The reference sample was labeled with Cy2. After labeling, samples were randomly paired and loaded on 24 cm immobilized pH gradient strips, pH range 3–10 nonlinear (GE Health Care, Piscataway, NJ). Labeled samples (50 μg) plus reference sample (50 µg) were mixed with unlabeled sample (450 μg) to give a total of 600 µg of protein and the volume was adjusted with sample buffer (8 M urea, 130 mM DTT, 4% (w/v) CHAPS, and 2% (v/v) ampholytes pH 3–10) to reach 450 µL. Strips were rehydrated overnight at room temperature with the protein mixtures. IEF was performed with an Ettan IPGphor3 system (GE Health Care, Piscataway, NJ) at 200 V for 30 min, 500 V for 30 min, and then ramped up to 10,000 V until 85,000 V hours was reached to ensure complete separation. To reduce and alkylate the proteins after the first-dimension separation, the strips were equilibrated for 15 min in equilibration buffer (6 M urea, 75 mM Tris–HCl, pH 8.8, 29.3% glycerol, 2% SDS, and 0.002% bromophenol blue) containing 2% DTT, followed by the addition of 200 mM iodoacetamide for a second incubation of 15 min. The strips were loaded onto 4–16% gradient, 24 cm SDS gels (Jule Biotechnologies Inc, Milford, CT) followed by electrophoresis at 10 W for 12 h.

The Typhoon Trio + Variable Mode Imager (GE Health Care, Piscataway, NJ) was used to obtain gel images with the following settings: 532 nm excitation laser and 580 BP 30 emission filter (Cy3), 633 nm excitation laser and 670 BP 30 emission filter (Cy5), and 488 nm excitation and 520 BP 40 emission filter (Cy2). DeCyder Differential Analysis Software v.7 (GE Health Care, Piscataway, NJ) was used to detect the differentially expressed proteins after normalizing to the reference sample. Following the automated search for differentially expressed proteins based on densitometry of the spots, the Genomic Solutions Investigator ProPic Robotic Workstation was used to excise gel plugs that contained differentially expressed proteins from the gel.

The excised gel plugs were digested with 50 µL of Promega sequencing grade trypsin solution (12.5 ng/µL in ammonium bicarbonate buffer, ABC) following standard procedures overnight. Peptides were extracted with 70% ACN + 0.1% TFA and injected onto a PepMap nanoflow HPLC column (75 µm i.d. × 15 cm) (LC Packing, C18) and chromatographed using a binary gradient (Solvent A, 3% ACN v/v and 0.1% acetic acid v/v) and Solvent B (97% ACN v/v and 0.1% acetic acid v/v) into an LTQ Orbitrap XL (Thermo Fisher, Waltham, MA, USA) mass spectrometer. The gradient started with 3% solvent B, ramped to 40% solvent B and held for 30 min^42^. The instrument was operated in data dependent mode with the Xcalibur 2.07 LTQ Orbitrap Tune Plus 2.55 software for the LC–MS/MS analysis. MS spectra were scanned in a range of m/z 300–2000 with 60,000 resolution at *m/z* 400. During collision induced dissociation (CID), the top five intensity ions were fragmented and a 60 s dynamic exclusion time was applied. The conditions of the MS run included a 2.2 kV spray voltage, 200 °C heated capillary temperature, 44 V capillary voltage, zero auxiliary gas and sheath flow, 165 V tube lens voltage, 1.0 m/z ion isolation width, 35% CID collision energy (normalized) during MS2, 500 count ion selection threshold, an activation q set to 25, and an activation time (T) of 30 ms. The results of the scan were compared against the manatee and mammalian databases (576,208 and 20,140,000 entries, respectively) and analyzed by Scaffold (version Scaffold-02-03-01, Proteome Software Inc., Portland, OR)^43^ and Mascot (Matrix Science, London; version 2.2.2)^44^ to scan the manatee genome and validate the peptides and proteins assigned. Fragment ion mass tolerance was set to 0.50 Da with a 15 ppm parent ion tolerance. The Peptide Prophet Algorithm accepted only peptides with > 95.0% probability in their assignments^45^. In the separate Protein Prophet algorithm, protein acceptance was based on > 99.0% probability and required that two or more unique peptides be identified^46^.

### iTRAQ

For relative quantifications of the proteins present in the manatee serum, 100 µg of total protein per sample was digested with trypsin and labeled using the iTRAQ Reagents 8-plex kit according to the manufacturer’s instructions (AB SCIEX, Inc., Foster City, CA)^47^. The manatees from the IRL and red tide groups were labeled with tags corresponding to 117, 118, 119, and 121 masses (Fig. 1) and each sample from the exposed groups was compared to the controls (labeled with tags corresponding to 113, 114, 115, and 116, Fig. 1), in two separate experiments.

Labeled samples were first fractionated by strong cation exchange chromatography using a polysulfoethyl A column (2.1 X 100 mm, 5 µm, 300 Ǻ; PolyLC, Columbia, MD, USA) with a binary gradient over 50 min of 0–20% solvent B with Solvent A (25% (v/v) ACN, 10 mM ammonium formate and 0.1% (v/v) formic acid (pH 2.8)) and solvent B (25% (v/v) ACN in 500 mM ammonium formate-pH 6.8)^42,48^. Ten fractions were collected, and each fraction was individually analyzed by reverse phase LC–MS/MS using a 15 cm nanoflow column (PepMap 75 µm id, 3 µm, 100 Å) with a 300 nL/min flow rate on a nanoLC ultra 1D plus system (AB SCIEX, Foster City, CA)^42^. The chromatography buffers were Solvent A (3% ACN v/v and 0.1% acetic acid v/v) and Solvent B (97% ACN v/v and 0.1% acetic acid v/v) and a gradient was used of solvent B (from 3–40%) for 120 min and then to 90% B for 5 min. The eluted peptides were directly sprayed into an LTQ Orbitrap XL mass spectrometer (Thermo Fisher, Waltham, MA). MS2 spectra were acquired in a data-dependent mode as described above for the 2D-DIGE experiment. An Orbitrap full MS scan was performed via HCD on the top 10 most abundant ions. The isolation window for ion selection was 2 m/z. Normalized collision energy was set at 35% and the dynamic exclusion time was 20 s.

Data from iTRAQ were input into ProteinPilot™ software version 4.0. The search in ProteinPilot™ was performed using a user-defined Paragon Algorithm to identify and quantify the proteins in the samples. Data were searched against the manatee and mammalian databases (576,208 and 20,140,000 entries, respectively)^42^, as described for the DIGE experiment. To calculate a false discovery rate (FDR) for peptide-protein assignments, Proteomics System Performance Evaluation Pipeline (Proteomics PEP, Applied Biosystems) in Protein Pilot was used to create a reversed database. Peptide identifications were accepted if they could be established at greater than 90% probability using the Scaffold Local FDR algorithm and protein identifications, if they could be established at greater than 95.0% probability and contained at least 2 identified peptides. Proteins were considered further as biomarkers if they were over expressed with an average fold change (FC) > 1.2-fold or under expressed by FC < 0.8-fold. The mass spectrometry proteomics data were deposited in the ProteomeXchange Consortium via the PRIDE partner repository with the dataset identifier PXD002470 and 10.6019/PXD002470^49,50^.

### Western blot methods

Western blots were performed on the 12 original manatee serum samples, IRL (n = 4), red tide (n = 4), and Crystal River controls (n = 4). For a positive control for antibody staining, a human blood sample was used that contained the proteins of interest. Molecular weight marker (SeeBlue Plus2) was added to the first lane. The samples were electrophoresed on 4–12% Bis–Tris Midi Gels from NuPAGE Novex (Thermo Fisher, Waltham, MA). The running buffer was 1X MES-SDS, as recommended by Novex. Gels were electroblotted to Turbo Blot 0.2 µm PDVF membranes in a Trans-Blot Turbo Transfer System (BioRad, Hercules, CA).

Western blots were developed by first blocking with 5% blotto in TBST (20 mM Tris, pH 7.5, 150 mM NaCl, 0.1% Tween 20) and then hybridized with the primary antibody of interest. Rabbit polyclonal antibodies against human proteins, complement C4 (C4 antibody [N1N2-2]), kininogen (rabbit anti-human kininogen 1 polyclonal antibody), ceruloplasmin (CP, ID (Ceruloplasmin, Ferroxidase)), and transthyretin (rabbit anti-human transthyretin polyclonal antibody), were obtained from MyBioSource (San Diego, CA) and diluted to 0.1 µg/ml and 3 µg total antibody was used per blot, as recommended by the manufacturer. The secondary antibody (Novex Goat anti-Rabbit IgG (H + L) Polyclonal Secondary Antibody, HRP conjugate) was added in a ratio of 1:1,000 for a 2-h incubation, after which Luminol reagent (Santa Cruz Biotechnology, Dallas, TX) was added to develop the western blots. A Gel Doc XR + System (BioRad, Hercules, CA) was used to view the results, relative to controls and ImageJ was used to quantify differences in protein concentrations^51^.

### Pathway analysis

Pathway Studio V9 (Elsevier, Alpharetta, GA), operating with the ResNet 9.0 database, was used to identify potential disease pathways for the red tide and IRL manatees compared to controls from Crystal River. Since it is likely that manatee proteins with high homology to their human counterparts may have similar functions, the differentially expressed proteins obtained from iTRAQ and 2D-DIGE were searched for the gene names of their homologs in the human databases and entered into Pathway Studio with their respective fold changes. Subnetwork enrichment analysis (SNEA) was performed using the Fisher’s Exact Test (p < 0.05) to search suspected disease related pathways^52^.

## Supplementary Information

Supplementary Information.

## Abbreviations

AGT: Angiotensinogen
A2M: Alpha-2-macroglobulin
AHSG: Alpha-2-HS-glycoprotein
ALB: Albumin
APCS: Amyloid P component, serum
APOA1: Apolipoprotein A–I
APOB: Apolipoprotein B
C1QA: Complement component 1, q subcomponent
F5: Coagulation factor V
C3: Complement component 3
C4A: Complement component 4A
C4BPA: Complement component 4 binding protein
C5: Complement component 5
CD5L: CD5 molecule-like
CFP: Complement factor properdin
CLU: Clusterin
CRP: C-reactive protein
F2: Coagulation factor II (thrombin)
FBLN2: Fibulin 2
FGA: Fibrinogen alpha chain
GC: Group-specific component (vitamin D binding protein)
GSN: Gelsolin
ITIH1: Inter-alpha (globulin) inhibitor H1
ITIH2: Inter-alpha (globulin) inhibitor H2
ITIH4: Inter-alpha (globulin) inhibitor H4
KNG1: Kininogen 1-like 1
LUM: Lumican
PLAT: Plasminogen activator
PROS1: Protein S
SERPINA4: Serpin peptidase inhibitor, clade A member 4 (antitrypsin)
SERPINC1: Serpin peptidase inhibitor, clade C (antithrombin)
SERPIND1: Serpin peptidase inhibitor, clade D1
SERPINF1: Serpin peptidase inhibitor, clade F1
THBS1: Thrombospondin 1
VTN: Vitronectin
