# Supplementary material for: Investigating an increase in Florida manatee mortalities using a proteomic approach
Source: Sci Rep. 2021 Feb 19;11:4282. doi: 10.1038/s41598-021-83687-y (PMC7895937; doi:10.1038/s41598-021-83687-y)
Supplement: Supplementary file 1 — Supplementary Information. [file 41598_2021_83687_MOESM1_ESM.pdf]

Supplementary Material

Investigating an Increase in Florida Manatee Mortalities Using a Proteomic Approach

Rebecca Lazensky[1,2], Cecilia Silva-Sanchez[3], Kevin J. Kroll[1], Marjorie Chow[3], Sixue Chen[3,4], Katie Tripp[5], Michael Walsh*[2], and Nancy D. Denslow*[1]

[1]Center for Environmental and Human Toxicology, University of Florida, Building 471 Mowry Road, Gainesville, FL, 32611, USA

[2]Aquatic Animal Health Program, College of Veterinary Medicine, University of Florida, PO Box 100136, Gainesville, FL, 32610, USA

[3]Proteomics and Mass Spectrometry, Interdisciplinary Center for Biotechnology Research, 2033 Mowry Rd, Gainesville, FL, 32610, USA

[4]Department of Biology, University of Florida Genetics Institute, Gainesville, FL, 32611, USA

[5]Save the Manatee Club, 500. N Maitland Ave., Maitland, FL, 32751, USA

Supplementary Figure 1.

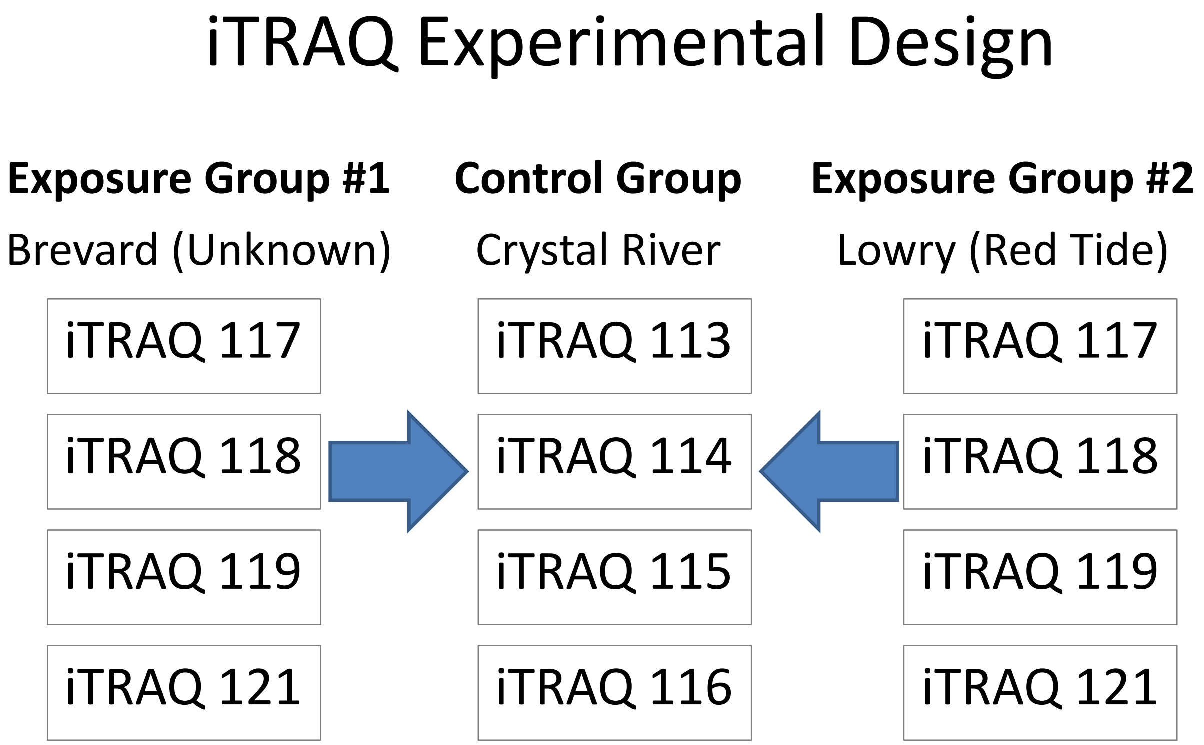

Supplementary Figure 1. Schematic showing how samples from each of the manatee groups were marked with iTRAQ labels. The exposure groups were (1) manatees collected from the Indian River Lagoon in December 2012, close to where there was an unknown mortality event, (2) manatees recovering from red tide at Zoo Florida and (3) manatees collected from Crystal River in February 2013 during annual health assessments and which served as controls with an N of 4 for each group.

Supplementary Figure 2. Gel and western blot images of the antibodies tested

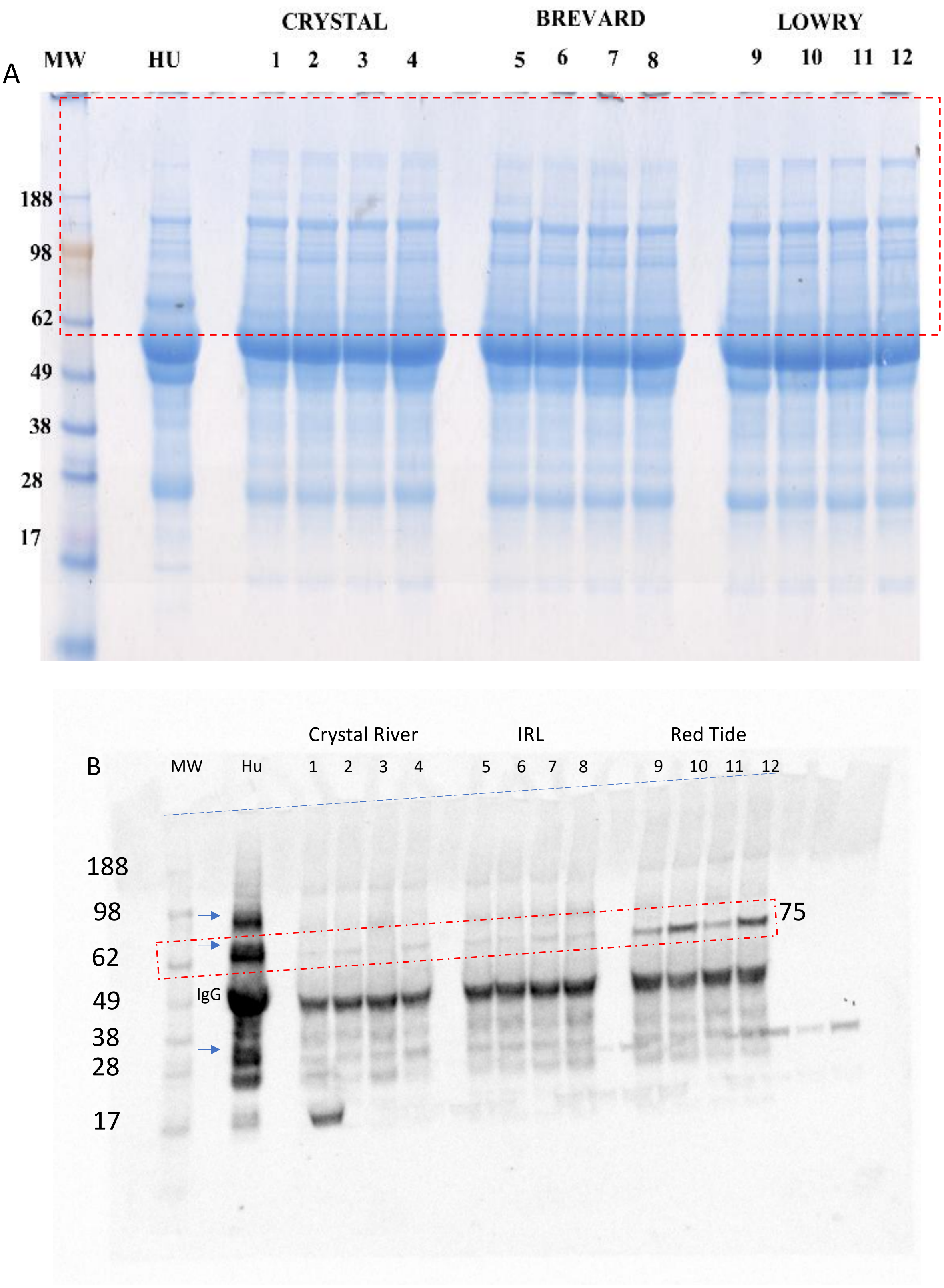

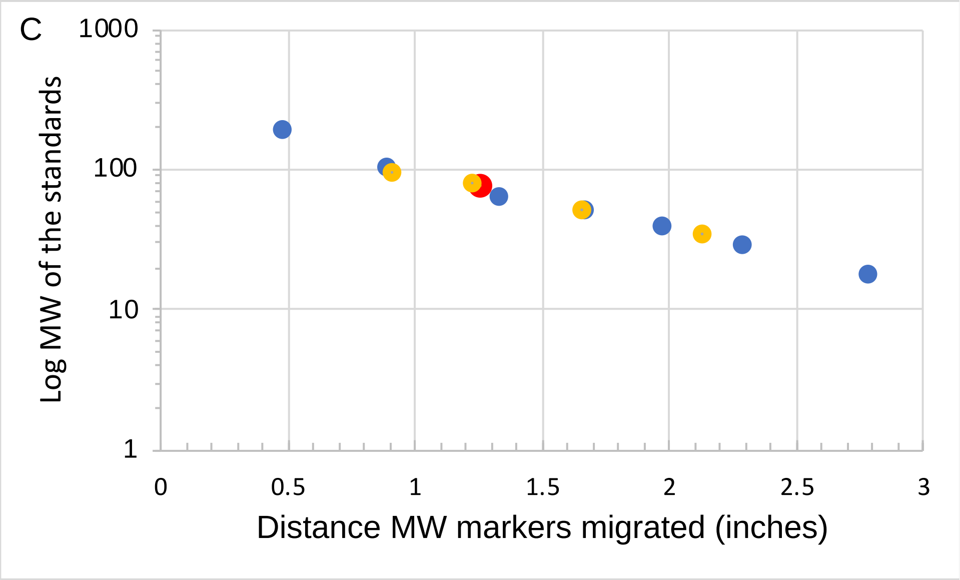

Supplementary Figure 2. Gel and Western blot images of serum obtained from manatees sampled at Crystal River, IRL and Red Tide. (A) Representative Coomassie blue stained gel showing that equal amounts of protein were loaded into the wells of the gel. HU, corresponds to a sample of human plasma that was electrophoresed in the gel at the same protein concentration as the samples to serve as a positive control for the C4 antibody. Red boxed area is area shown in Fig. 2 in the manuscript. (B) Western blot probed with C4 antibody [N1N2-2] obtained from MyBioSource (San Diego, CA). HU, corresponds to the human protein response (positive control). Human C4 protein is composed of three subunits with molecular weights of 95, 75 and 33 KDa (arrows) in the HU column. The large band at 50 KDa is IgG, which apparently also reacted with the antibody in a non-specific manner. The only manatee C4 subunit that cross reacted with the primary antibody was the 75 kDa subunit, shown on the left with an arrow. Red boxed area is area shown in Fig. 2 in the manuscript and it is tilted to match the tilt of the gel on the blot, illustrated by blue dashed blue line at top.. (C) Standard curve based on the MW of the standards (blue dots), the human C4 protein subunits (yellow dots) used for calculation of MW of manatee 75 KDa subunit of C4 (red dot).

Supplementary Table 1. Proteins identified in the 2D-DIGE experiment.

Proteins identified	Accession #	Theoretical molecular weight	# of unique peptides exclusively assigned[a]	# of spots in which protein was found
C-reactive protein-like*	gi|471417556	25 kDa	7	9
Complement C4-A isoform 1*	gi|471418672 (+1)	193 kDa	4	7
Serum albumin*	gi|471401421	70 kDa	10	5
Histidine-rich glycoprotein*	gi|471398263	64 kDa	6	4
Gelsolin*	gi|471370698	86 kDa	13	5
C-reactive protein-like*	gi|471417554	25 kDa	4	2
Coagulation factor V*	gi|471404036	232 kDa	6	3
Complement factor H-related protein 2*	gi|471369811	36 kDa	6	2
Complement C3*	gi|471381350	186 kDa	5	4
Kininogen-1 isoform 1*	gi|471397997	48 kDa	12	2
Vitronectin*	gi|471407831	53 kDa	3	2
C4b-binding protein alpha chain*	gi|471369763	102 kDa	4	1
CD5 antigen-like*	gi|471418788	38 kDa	6	1
EGF-containing fibulin-like extracellular matrix protein 1	gi|471357833	56 kDa	7	1
Alpha-2-macroglobulin*	gi|471411580	165 kDa	2	1
Angiotensinogen*	gi|471364912	53 kDa	9	1
Antithrombin-III isoform 1*	gi|471364683 (+1)	53 kDa	4	1
Apolipoprotein M	gi|471418612	21 kDa	2	1
Coagulation factor X	gi|471400300	42 kDa	2	1
Complement C1s subcomponent*	gi|471411546	77 kDa	9	1
Complement component C6*	gi|471413910	105 kDa	10	1
Creatine kinase M-type	gi|471395805	43 kDa	2	1
Myosin light chain 1/3, skeletal muscle isoform	gi|471375459	17 kDa	3	1

Peroxiredoxin-2	gi|471416562	22 kDa	3	1
Prothrombin*	gi|471412368	70 kDa	2	1
Tropomyosin alpha-4 chain isoform 1	gi|471368317 (+4)	37 kDa	2	1

* Proteins that were also identified in the iTRAQ experiment

[a]The number of unique peptides exclusively assigned to a protein (across all spots)

Supplementary Table 2. Serum proteins differentially expressed as determined by iTRAQ for Red tide exposed manatees

#	Name proteins	Average fold change ratio	[a]Peptides (95%)	[b]Unused score	[c]%Cov
1	Ceruloplasmin-like	2.32	24	25.07	47.39
2	Pyruvate kinase isozymes M1/M2 isoform 3	2.29	4	4.01	50.85
3	Angiotensinogen*	2.08	17	23.71	48.68
4	Complement C4-A isoform 1*	1.83	104	126.92	67.7
5	C-reactive protein-like*	1.68	20	17.59	49.55
6	Sulfhydryl oxidase 1	1.65	6	10.39	49.67
7	Vitronectin*	1.57	25	23.37	46.98
8	Complement C1q subcomponent subunit C	1.53	8	6.59	34.87
9	Heparin cofactor 2	1.5	10	16.09	43.46
10	N-acetylmuramoyl-L-alanine amidase	1.48	3	4.81	28.99
11	Complement C1q subcomponent subunit B	1.46	3	2.95	41.43
12	Complement C3*	1.42	107	119.27	71.81
13	Gelsolin*	1.41	13	18.8	43.13
14	Thrombospondin-1	1.36	9	15.36	42.31
15	Complement C1s subcomponent*	1.33	6	7.43	29.32
16	Fibulin-1 isoform 2	1.24	5	2.66	40.58
17	Kallistatin	1.18	2	4.15	30.14
18	Alpha-2-macroglobulin*	1.17	11	15.72	39.05
19	Properdin	1.15	2	3.95	28.66
20	Complement C5	1.14	12	26.23	42.53
21	Fibulin-1 isoform 1	1.14	7	12	42.41
22	Alpha-2-HS-glycoprotein	1.12	7	7.29	45.45
23	Complement C1q subcomponent subunit A	1.09	2	1.97	40.82
24	Alpha-2-antiplasmin	1.08	5	7.38	44.54

25	Inter-alpha-trypsin inhibitor heavy chain H4	1.08	8	8.46	32.32
26	Actin, cytoplasmic 2	1.06	4	6.07	51.73
27	Plasminogen-like isoform 2	1.06	8	13.08	46.84
28	Alpha-2-macroglobulin-like*	1.05	8	12.16	35.09
29	Serum amyloid P-component	0.96	11	14.49	54.26
30	Apolipoprotein B-100	0.94	35	61.87	40.93
31	Serum albumin*	0.92	16	27.06	68.8
32	Complement component C6*	0.89	4	8.55	42.66
33	Complement component C7	0.89	2	2.42	24.29
34	Vitamin K-dependent protein S	0.87	10	10.78	43.2
35	Apolipoprotein A-I	0.86	3	4.61	47.79
36	Serotransferrin	0.84	2	3.44	40.17
37	Coagulation factor V*	0.83	6	8.23	28.12
38	Complement component C9	0.83	2	4.84	33.33
39	complement factor H-related protein 2*	0.83	2	4	38.22
40	Clusterin isoform 2	0.82	32	22.52	60.04
41	Uncharacterized protein LOC101341768	0.81	2	3	22.63
42	Prothrombin*	0.8	29	42.48	66.67
43	C4b-binding protein beta chain	0.78	2	2.45	24.91
44	C4b-binding protein alpha chain*	0.77	44	40.59	55.21
45	CD5 antigen-like*	0.76	7	10.21	55.75
46	Vitamin D-binding protein	0.75	2	2.06	44.84
47	Inter-alpha-trypsin inhibitor heavy chain H2	0.71	5	6.13	26.98
48	Coagulation factor XIII A chain	0.7	2	4.1	22.44
49	Immunoglobulin J chain	0.7	3	4.32	39.24
50	Insulin-like growth factor-binding protein complex acid labile subunit	0.7	3	5.64	39.59
51	Antithrombin-III isoform 1*	0.69	32	46.05	77.63

52	Lumican	0.69	2	3.59	19.41
53	Inter-alpha-trypsin inhibitor heavy chain H3	0.67	7	12.71	34.43
54	Alpha-1B-glycoprotein	0.65	5	8.51	39.15
55	Fibrinogen alpha chain	0.62	3	5.25	38.58
56	Fibronectin isoform 3	0.57	47	62.96	47.04
57	Inter-alpha-trypsin inhibitor heavy chain H1	0.57	2	4.34	31.9
58	Histidine-rich glycoprotein*	0.56	17	22.98	45.31
59	Kininogen-1 isoform 1*	0.54	11	13.46	59.81
60	Transthyretin	0.54	2	4.46	41.5

*Proteins that were also identified in the 2D-gel electrophoresis experiment.

a, peptide (95%) refers to the number of peptides belonging to the protein that were identified with > 95% confidence in the sequence.

b, unused score is a score that is related to the probability that the assignment of a peptide to a protein is correct in the ProteinPilot™ software. The more a protein has peptides exclusively assigned to it, the more likely the protein assignment made is correct. An unused score threshold of 1.3 or above assures 95% confidence in the results.

c, % Cov, is the percentage coverage of the protein from the peptides that were identified.

Supplementary Table 3. Serum proteins differentially expressed as determined by iTRAQ for manatees sampled in the IRL

#	Name (predicted proteins)	Average ratio	[a]Peptides (95%)	[b]Unused score	[c]% Cov
1	Kininogen-1 isoform 1*	1.38	19	27.56	78.97
2	Protein AMBP	1.38	2	2.04	27.71
3	Histidine-rich glycoprotein*	1.34	19	28.17	54.87
4	Properdin	1.3	2	3.26	26.72
5	Complement C4-A isoform 1*	1.25	94	118.91	65.23
6	Vitronectin*	1.25	20	21.43	47.2
7	Ceruloplasmin-like	1.22	19	25.52	45.38
8	Alpha-2-HS-glycoprotein	1.22	11	11.65	38.57
9	Inter-alpha-trypsin inhibitor heavy chain H2	1.21	14	23.93	40.32
10	Heparin cofactor 2	1.18	9	16.4	55.73
11	Vitamin K-dependent protein S	1.18	9	14.93	48.9
12	Alpha-2-macroglobulin-like*	1.12	12	20.94	38.67
13	Glutathione peroxidase 3, partial	1.11	2	2.91	52.78
14	Complement C3*	1.09	102	133.13	72.05
15	Thrombospondin-1	1.09	10	20.42	41.71
16	C4b-binding protein alpha chain*	1.08	51	50.9	60.95
17	C-reactive protein-like*	1.07	19	14.3	50
18	Kallistatin	1.07	3	6.09	31.78
19	Alpha-1B-glycoprotein	1.06	11	16.72	49.15
20	Fibulin-1 isoform 1	1.06	7	8.33	41.84
21	C4b-binding protein beta chain	1.06	3	4.59	37.17
22	Plasminogen-like isoform 2	1.05	12	19.68	54.4
23	Coagulation factor VII	1.05	2	4	37.86
24	Antithrombin-III isoform 1*	0.96	38	49.71	79.78
25	Inter-alpha-trypsin inhibitor heavy chain H4	0.96	10	10.7	40.68
26	Alpha-2-antiplasmin	0.96	6	11.35	38.97

27	Actin, cytoplasmic 2	0.93	5	7.97	60.53
28	Lumican	0.92	5	7.28	45.59
29	Complement factor H-related protein 2*	0.92	5	6.04	39.17
30	Fibulin-1 isoform 2	0.91	6	2	33.87
31	Insulin-like growth factor-binding protein complex acid labile subunit	0.91	3	6.13	44.01
32	Gelsolin*	0.9	12	15.57	44.4
33	Fibrinogen alpha chain	0.9	3	6.37	32.33
34	Angiotensinogen*	0.89	11	20.71	50.92
35	Coagulation factor V*	0.89	10	16.53	33.19
36	Apolipoprotein A-I	0.89	5	8.22	58.82
37	Sulfhydryl oxidase 1	0.89	4	8.44	42.09
38	Alpha-2-macroglobulin	0.88	11	20.34	31.96
39	Coagulation factor XIII A chain	0.88	3	5.18	34.5
40	Serum amyloid P-component	0.87	10	18.04	59.19
41	Immunoglobulin J chain	0.87	5	6.36	51.9
42	Titin	0.87	2	2.44	24.03
43	Serum albumin*	0.85	15	27.24	63.55
44	Vitamin D-binding protein	0.85	3	3.84	50.09
45	Fibronectin isoform 3	0.83	63	81.34	55.57
46	CD5 antigen-like*	0.83	10	17.27	56.61
47	N-acetylmuramoyl-L-alanine amidase	0.83	2	4.5	44.43
48	Transthyretin	0.7	2	4.93	54.42

*Proteins that were also identified in the 2D-gel electrophoresis experiment.

a, peptide (95%) refers to the number of peptides belonging to the protein that were identified with > 95% confidence in the sequence.

b, unused score is a score that is related to the probability that the assignment of a peptide to a protein is correct in the ProteinPilot™ software. The more a protein has peptides exclusively assigned to it, the more likely the protein assignment made is correct. An unused score threshold of 1.3 or above assures 95% confidence in the results.

c, % Cov, is the percentage coverage of the protein from the peptides that were identified.
